# The Story of Us: Older and Younger Couples’ Language and Emotional Responses to Jointly Told Relationship Narratives

**DOI:** 10.1093/geroni/igaa057.1854

**Published:** 2020-12-16

**Authors:** Stephanie Wilson, William Malarkey, Janice Kiecolt-Glaser

**Affiliations:** 1 Southern Methodist University, Dallas, Texas, United States; 2 The Ohio State University College Of Medicine, columbus, Ohio, United States

## Abstract

Social-emotional well-being is said to improve with age, but evidence for age differences in couples’ behavior and emotions—studied primarily during marital conflict—has been mixed. Characteristics of jointly told relationship stories predict marital quality among newlyweds and long-married couples alike, yet younger and older couples’ accounts have never been compared. To examine age differences in couples’ emotional responses and in their I/we-talk, emotion word use, and immediacy (i.e., self-focused, present-tense style), 42 married couples ages 22–77 recounted their relationship’s history then rated the discussion and their moods. Compared to younger couples, older couples used more we than I language, more positive than negative words, and less immediacy. Partners in older pairs shared more similar language patterns. In turn, lower immediacy mediated links between older age and less negative mood, and explained husbands’ more positive appraisals. Indeed, relationship accounts reveal novel insights into age differences in marriage and well-being.

